# Viroimmunotherapy for Colorectal Cancer:  Clinical Studies

**DOI:** 10.3390/biomedicines5010011

**Published:** 2017-03-10

**Authors:** Shyambabu Chaurasiya, Susanne Warner

**Affiliations:** Beckman Research Institute, City of Hope National Medical Center, Duarte, CA 91010, USA; schaurasiya@coh.org

**Keywords:** oncolytic virus, colorectal cancer, immunotherapy, viral gene therapy

## Abstract

Colorectal cancer is a leading cause of cancer incidence and death. Therapies for those with unresectable or recurrent disease are not considered curative at present. More effective and less toxic therapies are desperately needed. Historically, the immune system was thought to be an enemy to oncolytic viral therapy. Thinking that oncolysis would be the only mechanism for cell death, oncolytic virologists theorized that immune clearance was a detriment to oncolysis. Recent advances in our understanding of the tumor microenvironment, and the interplay of tumor survival and a patient’s immune system have called into question our understanding of both arenas. It remains unclear what combination of restrictions or enhancements of innate and/or cell-mediated immunity can yield the highest likelihood of viral efficacy. This article reviews the variety of mechanisms explored for viruses such as immunotherapy for colorectal cancer.

## 1. Introduction

The human immune system has evolved for eons to respond to exogenous infectious organisms like bacteria, fungi, and viruses. Likewise, organisms like viruses have evolved to evade host immune systems. Cancer cells, while historically poorly understood, have a robust ability to evade immune cell destruction. Recently, researchers in the silos of oncolytic viral therapy and cancer immunotherapy have begun to merge the principles of their respective fields, and seek to understand how oncolytic viruses can incite and/or train the host immune system against tumors. Colorectal cancer is the third most common cancer and the third leading cause of cancer death in the United States [1]. Despite enhanced survival across all stages with recent improvements in targeted chemotherapy and aggressive surgical management, patients suffering distant metastases still have a dismal survival of under 15% [1]. Novel therapies are desperately needed to prevent and treat primary colorectal cancer and metastases. This article explores the complex interplay between the available data regarding viroimmunotherapy for colorectal cancer.

Early in the evolution of the field, oncolytic viral therapy researchers focused on oncolysis as the most predominant form of tumor cell killing. Thus, initial efforts were aimed at immunosuppression to prevent viral clearance and allow for increased viral oncolysis. However, as virotherapy and immunotherapy have evolved, investigators have begun to elucidate the complex interactions of viruses, the immune system, and the tumor microenvironment. Unfortunately, given the complexities at hand, the literature contains conflicting information and theories wherein expectations fail to match up with reality [2,3]. For instance, one would expect that viral efficacy in immunodeficient animal models would be dampened in immunocompetent models, and while some data would support this theory [4,5], other authors have shown that this does not always prove to be true [6]. Moreover, one would expect that some component of oncolysis or viral infection would be needed to initiate an immune response, yet elegant data wherein viral agents are unable to infect certain tumor models still achieve anti-tumor effects by initiating a natural killer response [7]. It is likely that some combination of immunosuppression to block viral clearance, facilitation of innate immune activity against tumor cells, and augmentation of humoral immunity to facilitate long term anti-tumor immunity will ultimately yield success. Investigators have taken many different angles to address these questions. From viruses as cancer vaccines to engineered immune cells in combination with viral therapies, there are a number of different approaches that can shed light on some of the fundamental principles of viroimmunotherapy. This article reviews and contextualizes the available preclinical and clinical data regarding immunotherapy and viral immunotherapy for colorectal cancer.

## 2. Cancer Vaccines

The context of oncolytic viral immunotherapy cannot be fully understood without understanding the groundwork laid in the standard immunotherapy community up to this point. Recent studies have pointed out that the response of a tumor to immunotherapy depends on the immunogenicity of the tumor [8]. Certain types of cancer such as metastatic melanoma and renal cell carcinoma are thought to be highly immunogenic based on the following: (i) occasional spontaneous regression [9,10]; (ii) improved survival associated with infiltrating T-lymphocytes [11,12]; (iii) response to non-antigen specific immunotherapies such as interferon-α, interleukin-2, and anti-cytotoxic T-lymphocyte-associated antigen 4 (CTLA4) [13,14]; (iv) higher incidence of these malignancies in immune-suppressed individuals [13,15]; and (v) presence of tumor-associated antigens and human leukocyte antigen (HLA)-restricted epitopes within these antigens [16,17]. Until recently, colorectal cancer was thought to have low immunogenicity, and was considered a poor candidate for immunotherapy [18]. However, recent studies have shown that the genetic and epigenetic changes contributing to the development of colorectal cancer also result into the formation of neo-antigens that are recognised by the immune system [19,20,21]. These neo-antigens, tumor-associated antigen (TAA), or tumor-specific antigen (TSA), have been shown to elicit anti-tumor immune response. Furthermore, tumor infiltration by immune cells has been shown to correlate with better prognosis in colorectal cancer (CRC) patients [22]. These findings suggest that CRC could be an excellent target for immunotherapy.

The ultimate goal of a therapeutic cancer vaccine is to eliminate the existing tumor and prevent cancer recurrence. Cancer vaccines are inherently difficult to develop because they require an appropriate antigen target, and a robust understanding of immune response and how to manipulate it [23]. Thus, it is assumed that each tumor or tumor cell possess a uniform quality, and that an immune system trained against a single antigen will accomplish eradication. In theory, by infecting cancer cells and targeting immunity against infected cells, the virus-mediated immunity subverts the need for tumor-specific antigen. However, since it is highly unlikely that every tumor cell will get infected with the virus, the use of virus alone would not be sufficient to eliminate tumors. Investigators have tried a variety of vaccine strategies including tumor cells, peptides, dendritic cells, DNA, and viral vector-based vaccines [24].

## 3. Virus Infected Autologous Tumor Cell Vaccines

One interesting combination of principles came from the employment of virus-infected irradiated tumor cells as autologous cancer vaccine. Schlag et al. used colorectal cancer cells isolated from liver metastases for the vaccination purpose [25]. In this study, cells harvested from resected metastasized tumors were growth-arrested through irradiation. The cells were then infected with the Newcastle disease virus (NDV) and were injected into patients, a total of five times in two week intervals. This study found that the vaccination was well tolerated and there was a reduction in the rate of disease recurrence in vaccine-treated patients compared to the control group. A similar study by Ockert et al. compared the anti-tumor efficacy of autologous cancer cells that were infected with either NDV or bacillus Calmette-Guerin (BCG) [26]. In this study, the NDV-infected autologous cancer cells showed better anti-tumor response than the BCG-infected cells. The authors concluded that the presence of virus-encoded antigens on the surface of the infected cancer cells could have resulted in stronger anti-tumor immune response. Furthermore, the virus itself can stimulate different immune cells including CD4+ T cells, natural killer (NK) cells, and macrophages, all of which can mount an anti-tumor response [26]. Although these initial studies using NDV as adjuvant in autologous cancer cell vaccinations for colorectal cancer showed promising results, a recently completed large scale randomized trial failed to demonstrate the ability of such cancer vaccines to improve overall survival of CRC patients [27].

## 4. Viral Vector-Based Vaccines

Viruses are the most commonly used vectors for vaccination as they are inherently immunogenic [28,29]. Many types of viruses have the ability to directly infect dendritic cells (DC), the professional antigen presenters. Direct infection of DC by a virus engineered to express TSA or TAA could allow for enhanced presentation of tumor antigens to the T and B cells that could efficiently target the cancer cells [29,30]. Because of the ability of cancer cells to undergo ‘immune-edition’, they may escape the immune cells targeted to a single antigen [24,31]. Therefore, it is desirable to express multiple TAA and TSAs from the vector in order to minimize the risk of immune escape. However, every viral vector has a limited cloning capacity; some viruses may not be able to accommodate the gene for more than one antigen depending on the size of the antigen-encoding gene [32]. Therefore, viral vector should be selected based on several factors including but not limited to cloning capacity, immunogenicity, and pre-existing immunity.

Many viral vector-based therapeutic vaccines have been evaluated for colorectal cancer in pre-clinical studies, and several of them have been studied in different phases of clinical trial. These studies have used a variety of viruses including; poxvirus (vaccinia virus, canarypox virus, fowlpox virus, avipoxvirus, and modified virus Ankara); adenovirus; adeno-associated virus; and retroviruses [33]. These studies have explored many different proteins that are either exclusively or pre-dominantly expressed on colorectal cancer cells (CRC), as potential tumor-specific antigens. The most commonly targeted tumor antigens for therapeutic vaccinations of CRC include carcinoembryonic antigen (CEA), epithelial glycoprotien (Ep-CAM), and guanylyl cyclase 2C (GUCY2C) [34]. Table 1 shows a list of viral vectors encoding different tumor antigens that have been studied in clinical settings as therapeutic vaccines for CRC. Despite showing excellent safety profiles, these viral vectors have fallen short of showing meaningful anti-tumor effects in large scale clinical trials [35,36]. Failure of these vaccines may have been due, in part, to immune-edition by tumor cells as most of the viral vectors were engineered to encode only a single tumor antigen. Furthermore, the vectors completely relied on the immune system to eliminate tumors, as they were not intended for direct killing of cancer cells.

## 5. Oncolytic Virus

Unlike viral vector-based vaccines, oncolytic viruses (OVs) are designed to directly kill cancer cells by the virtue of their selective replication in cancer cells. Oncolytic viruses are thought to exert their anti-neoplastic activities through a variety of ways. While the exact mechanism of oncolysis differs from virus to virus, and even for the same virus depending on the structure and encoded transgene, there are some common mechanisms employed by most oncolytic viruses to achieve an anti-neoplastic effect. First, replication of many different viruses in a cancer cell can induce lysis of the cell [37]. Second, oncolytic viruses could induce specific and non-specific anti-tumor immunity that can aid to the overall efficacy of the virus. Although the role of immune system has been a matter of debate for a long time in oncolytic virotherapy, recent advancements suggest that the immune system plays a favorable role [38,39]. Oncolytic viruses are often constructed to encode a transgene that can further enhance the anti-tumor effect. A variety of transgenes ranging from immune-stimulatory genes to pro-apoptotic genes have been inserted into different oncolytic viruses to enhance their anti-tumor efficacy. For example, the immune-stimulatory genes IL-2, IL-4, IL-12, and granulocyte macrophage colony-stimulating factor (GM-CSF), and pro-apoptotic genes such as tumor necrosis factor α, p53, and tumor necrosis factor related apoptosis inducing ligands have been studied as therapeutic genes in different oncolytic viruses [40,41,42,43,44,45].

Cell death caused by direct replication of oncolytic viruses is complex and does not clearly fit into anyone of the traditional modes of cell death such as apoptosis, necrosis, or autophagy [46]. This is partly because oncolytic viruses are thought to hijack the cell death machinery, allowing death to occur only when cellular resources have been fully exploited for maximal production of progeny viruses [46]. Several studies have shown that cell deaths caused by oncolytic viruses are “immunogenic” [47,48,49]. A recent study by Tomoki et al. has shown that an oncolytic adenovirus causes immunogenic cell death in CT26 cells, and that the resulting lysate could protect immune-competent mice from tumor formation upon re-challenge with CT26 cells [50]. Several oncolytic viruses in combination with chemotherapeutic agents are currently being evaluated in clinical trials for the treatment of CRC. For example, the oncolytic vaccinia virus Jx-549 in combination with irinotecan has recently completed phase I trial in patients with CRC; the result of this study is yet to be published (NCT01394939). Likewise, Reolysin, an oncolytic reovirus, is also being evaluated in combination with irinotecan, leucovorin, 5-fluorouracil, and bevacizumab for the treatment of K-RAS mutant metastatic CRC (NCT01274624).

## 6. Combination of Oncolytic Viruses with Immune Checkpoint Inhibitors

Among many immune checkpoint proteins, programmed death 1 (PD-1) and CTLA4 are primarily employed by cancer cells to dampen anti-tumor immune response [51,52]. Consequently, inhibitors of CTLA4 and PD-1 have been shown to exert effective anti-tumor activity against a variety of malignancies in preclinical and clinical studies [51,53]. Ipilimumab, a monoclonal antibody against CTLA4 was the first checkpoint inhibitor to receive FDA approval [54]. It was approved in 2011 for the treatment of melanoma. Since then, several other checkpoint inhibitors have been approved, and many others are awaiting approval for the treatment of different malignancies [54,55]. Unfortunately, checkpoint inhibitors have not shown robust clinical efficacy in the treatment of colorectal cancer [56,57]. A recently completed phase II trial reported that tremelimumab, a fully humanised antibody against CTLA4, was unable to achieve objective responses in metastatic colorectal cancer patients [58]. Out of 45 evaluable patients, only one patient showed partial response, while the remaining patients all had progressive disease. Similarly, antibodies targeting the interaction between PD-1 and its ligand PD-L1 have shown little therapeutic benefits in unselected colorectal cancer patients [59]. Interestingly, PD-1 inhibitors have shown encouraging results against a small fraction of CRC patients with microsatellite unstable tumors [60]. Approximately 15% of all colorectal cancer cases have microsatellite instability, and previous studies have shown that microsatellite instability makes the tumor more immunogenic [61,62,63].

Recent studies have indicated that immune-activation is an important aspect in determining the anti-tumor efficacy of oncolytic viruses [39]. However, tumors often suppress the anti-tumor immune response especially through the checkpoint axis [64]. Therefore, it seems logical that the combination of the oncolytic virus with checkpoint inhibitors would result in more effective treatment of cancer. One of the earliest studies combining an oncolytic virus with checkpoint inhibitors was the combination of an oncolytic vesicular stomatitis virus (VSV) with anti-CTLA4 [65]. In this study, the combination resulted in complete regression of breast tumors in 80% of mice, while the combination of the same oncolytic virus with antibody against TGF-β or IL-10 resulted in complete tumor regression in less than 20% mice. Likewise, in 2014, a study by Zamarain et al. showed that the overall anti-tumor efficacy of an oncolytic NDV could be enhanced by combination with checkpoint inhibitors [66]. In this study, the authors used mouse models of melanoma (B16 cells) and colon cancer (MC38 cells) to investigate the therapeutic efficacy of an oncolytic NDV in combination with an anti-CTLA4 antibody. A combination of intra-tumoral injections of the oncolytic virus and systemically delivered anti-CTLA4 was found to be superior in controlling both injected and un-injected tumors compared to treatment with either the virus or anti-CTLA4 alone. The therapeutic effect of the combination therapy was dependent on tumor infiltration by CD8+ and CD4+ T cells, NK cells, and levels of type I interferon, and it was less dependent on the sensitivity of the cancer cells to NDV-mediated lysis. This study once again highlighted the importance of the immune system in the success of oncolytic viruses. Another study by Rojas et al. demonstrated that the combination of an oncolytic vaccinia virus with a CTLA4 inhibitor enhances anti-tumor response in mouse models of colon (MC38 cells) and renal (RENCA cells) cancer [67]. These pre-clinical studies have provided a strong rationale for testing the combination of oncolytic viruses with checkpoint inhibitors in clinical settings. Indeed, several early stage clinical trials are ongoing to evaluate the combination of oncolytic viruses with checkpoint (mainly PD-1 and CTLA4) inhibitors in a wide range of solid tumors. One such combination, that includes colorectal cancer patients, is the combination of enadenotucirev (a chimeric adenovirus) with nivolumab (anti-PD-1 antibody) in phase I clinical trial (NCT02636036).

## 7. Combination of Oncolytic Viruses with T Cells Expressing Chimeric Antigen Receptor

Like oncolytic viruses, T-cells engineered to express chimeric antigen receptors (CAR-T), are a novel class of experimental therapeutics. In preclinical and clinical studies CAR-T cells have shown very exciting results, especially in the treatment of hematological malignancies [77,78]. In contrast, for hematological malignancies, the effect of T cells in solid cancer has been modest, at best [79,80]. The poor performance of CAR-T cells in solid tumors is thought to be due, in part, to the immune-suppressed microenvironment in solid tumors and sub-optimal migration of the CAR-T cells to the tumors [81]. In order to ensure that high numbers of CAR-T cells reach target tumors, usually very high numbers of (up to 10 billion) CAR-T cells are injected into patients [81,82]. Such high doses of these cells often result into serious toxicities and could even be fatal. For instance, a colon cancer patient with metastatic lesions in their lungs and liver was injected intravenously with 1 × 10^10^ CAR-T cells expressing receptor for ERBB2 (an antigen over-expressed on many solid cancers including colon cancer). The patient suffered respiratory distress and cytokine storm, and ultimately succumbed to the treatment-related toxicities [82]. Similarly, in a separate study, three colorectal cancer patients were injected with 2–4 × 10^8^ CAR-T cells targeted to CEA; while only one patient showed partial response, all three patients experienced severe inflammatory colitis [83]. Despite having immense therapeutic potential, as evidenced from the trials with hematological malignancies, the usage of CAR expressing T-cells (CAR-T) cells in the treatment of solid cancers such as colorectal cancer has been plagued by the toxicities associated with high doses of these cells [80,81]. One way to bypass the severe toxicities would be to combine CAR-T cells with other therapeutics, ones that could potentially mitigate factors limiting the efficacy of CAR-T cells and allow achievement of meaningful anti-tumor response with low numbers of T cells.

**Figure 1:**
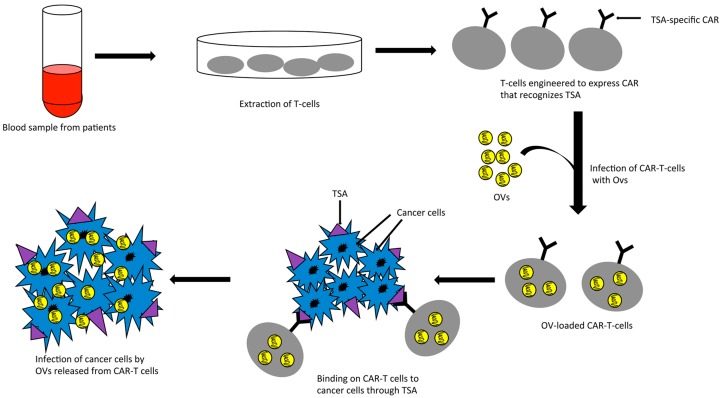
CAR-T cells as carrier for oncolytic viruses. TSA: tumor specific antigen; CAR: chimeric antigen receptor; OVs: oncolytic viruses; CAR-T: CAR expressing T-cells.

Interestingly, while the optimal anti-tumor activities of oncolytic viruses, especially in the face of preexisting antiviral immunity, are often limited by induction of immunity, the anti-tumor activities of CAR-T cells are often limited by an immune-suppressed environment within solid tumors. The opposing roles of the immune system on these two therapeutic agents suggest that a combination of CAR-T cells and oncolytic viruses may be complementary, and use the opposing roles of the immune system to their advantage. Indeed, a combination of an oncolytic adenovirus, encoding the cytokines IL-15 and RANTES, with CAR-T cells targeted to GD2 antigen has shown better anti-tumor efficacy than either of the treatments alone in a mouse model of neuroblastoma [84]. Another elegant strategy of combining oncolytic virus with CAR-T cells is the “Trojan horse” approach in which the CAR-T cells could be used as a carrier to safely deliver oncolytic viruses to the target tumors where both the virus and the T cells could wage the war against tumor cells (Figure 1). The use of CAR-T cells should protect the virus from antibody-mediated neutralization and anti-viral cellular immunity in the blood stream. The feasibility of such an approach was first demonstrated by Thorne et al. in a study where they used “cytokine induced killer” (CIK) cells as the carrier for delivering an oncolytic vaccinia virus to tumors through systemic routes in a mouse model [85]. Although they did not use CAR-T cells in their study, the concept of their study could be extrapolated for CAR-T cells as CIKs show inherent characteristics of tumor homing and cancer cell killing [86]. In line with this, a recent study by VanSeggelen et al. shows that oncolytic viruses with either RNA or DNA genome can be loaded onto CAR-T cells and efficiently transferred to tumor cells [87]. The anti-tumor effect of these novel combinations of oncolytic virus and CAR-T cells is yet to be evaluated in case of colorectal cancer.

## 8. Immune Analysis in Clinical Trials Examining Oncolytic Virus Versus Colorectal Cancer

Many oncolytic viral clinical trials have taken place in recent years. Few of them have been focused exclusively on colorectal cancer, and even fewer have gathered comprehensive if any information regarding immune response to viral therapy outside of the aforementioned trials utilizing viral vaccine vectors. Table 2 details trials of oncolytic virus versus colorectal cancer. While some of the more heterogeneous trials did include occasional colorectal cancer patients, their results did not include colorectal patients as an isolated group and have thus been excluded from this discussion. Much of our understanding of immune response to viral therapy is speculative. Because equipoise is hard to prove in patients with resectable diseases, most oncolytic viral trials involve patients who have failed multiple lines of traditional therapies and are not surgical candidates. For this reason, it is difficult to justify any tissue procurement. We are thus left with serum markers of immune reactivity and speculation.

In 2006, Kemeny and Fong detailed their experience injecting NV1020, an herpes simplex virus-1 (HSV-1) vector, via hepatic arterial infusion pumps. They found that fluctuation in IL-2, IFN-γ, and TNF-α were minor and did not exhibit a consistent pattern in relation to virus administration. Furthermore, CD4+ and CD8+ ratios varied inconsistently and in minor ways [88]. They saw impressive disease stability and responses in the highest dosing groups, but unfortunately, we do not glean much information in the way of immune response from this trial because tissue biopsies were not routinely performed.

Park et al. released their findings following administration of JX-549, an oncolytic and immunotherapeutic vaccinia virus expressing GM-CSF and β-galactosidase [89]. They found that GM-CSF was induced acutely after each infusion, though it was unclear if this was an endogenous expression, or an early expression resulting from viral infection [89]. The dose escalation involved four total injections. Eleven of the 15 trial patients completed all four planned injections. The remaining four patients left the trial early, secondary to disease progression. Interestingly, they found that certain collections of cytokines were elevated after the first doses, and lowered after the second doses (IL-6, IL-8, IL-18, macrophage inflammatory protein-1α, monocyte chemoattractant protein-1, MIP-1β, and TNF-α). This in contrast to a separate group which were higher after cycles 2–4 than after cycle one (IL-2, IL-10, IFN-γ). While there did appear to be dose-dependent tumor size stability in this study, no tumor tissue was obtained. That being said, this is one of the more comprehensive cytokine profile analyses and may serve as a foundation for later understanding of immune responses to vaccinia therapy [89].

Balint et al. evaluated a CEA-targeting adenovirus vaccine and specifically sought to analyze immunogenicity. They specifically examined cytolytic T cell responses, T-regulatory (Treg) and T-effector cell ratios in the context of HLA-A2 status. They noted a dose-dependent increase in CEA-specific cell-mediated immunity (CMI) with the majority of patients in the highest dose cohort experiencing CMI as indicated by high levels of IFN-γ secreting spot-forming cells [90]. They also showed significantly elevated granzyme B secretion post-immunization compared to baseline samples. This did not appear to change based on presence of HLA-A2. It does not appear that there was a consistent change in Treg to T-effector cell ratios over the treatment course [90]. They further noted that a high level of cell-mediated immunity as measured by IFN-γ secretion was required before detection of activated T cells.

Of note, in 2002, Pecora et al. administered PV701, a Newcastle Disease Virus to 79 patients, of whom 23 had colorectal cancer. They reported cytokine data for what they called 10 representative patients who were given one or more doses of 12 × 10^9^ or 24 × 10^9^ PFU/m^2^. In these patients, they measured IFN-α, IFN-β, IFN-γ, IL-6, and TNF-α and they did not stratify their results by disease type. They did however observe that IFN-α was the predominant cytokine produced and that detectable increases in all but IFN-β were seen within 6 h of dosing, usually peaking around 20 h and returning to baseline 2–3 days after dosing. Conversely, they reported that IFN-β was only detectable 20 h after dosing [91]. This is another demonstration that the focus of early oncolytic therapies during clinical trials was not on the complex interplay of immune mediators. Thus, it is difficult to draw meaningful conclusions about viroimmunotherapy from these data.

Calvo et al. have preliminarily reported data from their phase I dose-escalating study of an oncolytic Ad11/Ad3 chimeric group B adenovirus given intravenously to patients with metastatic epithelial tumors [92]. They report results from 34 patients, 26 of whom have colorectal disease. The virus was administered 3 days in a row as a single cycle. They report significant increase in cytokines (TNF, IFN, IL6, IL12) occurring on days in higher dosing groups, that attenuates with day 3 and 5 dosing and prolonged infusion duration. Further expanded results are awaited, but thus far no major immunologic conclusions have been published. Another trial looking at a reovirus vector combined with FOLFIRI and bevacizumab in FOLFIRI-naïve patients with KRAS mutant metastatic colorectal cancer is also being administered, and the oncolytic viral community eagerly awaits these results.

## 9. Conclusions

Oncolytic viroimmunotherapy for colorectal cancer is a rapidly evolving concept bridging two rapidly evolving fields. It is likely that some combination of the therapies listed above will ultimately be employed. However, in the meantime it is important for researchers in the field to more comprehensively address cytokine changes in the context of innate and cellular immunity. Designing trials that facilitate treated tumor procurement would be ideal correlates for the serum analyses. The coming decade is likely to bring a wealth of new information. It will be important for immunologists, virologists, oncologists, and surgeons to work closely together to advance the field and improve survival for colorectal cancer patients. 

## Figures and Tables

**Table 1:** Clinical trials that are using viruses as direct vaccines or as vectors in the preparation of autologous cell-based vaccines.

Virus	Treatment Type	Transgene (Tumor Antigen or Cytokine)	Phase of Trial	Outcome	Immune Response	References
Retrovirus	Therapeutic vaccination	IL-2	I	No objective response demonstrated	Tumor-specific CTL	[68]
Vaccinia virus	Therapeutic vaccination	CEA	I	No objective response	Not reported	[69]
Adenovirus	Therapeutic vaccination	CEA	I	Increased overall survival	CEA-specific immunity	[70]
Adenovirus	Therapeutic vaccination	GUCY2C	I	Not published	GUCY2C-specific antibody and T-cell responses	NCT01972737
Baculovirus	Therapeutic vaccination	Ep-CAM	I	Not published	Ep-CAM-specific cellular immune response	[71]
Canarypox virus	Therapeutic vaccination	Ep-CAM	I	Not published	Ep-CAM-specific cellular immune response	[72]
Avipox virus	Therapeutic vaccination	CEA, B7-1	Pilot	Stable disease in some patients	CEA-specific CTL	[73]
Vaccinia + Avipox virus	Therapeutic vaccination	CEA	I	No objective anti-tumor response	Antibody against CEA	[74]
Vaccinia + Fowlpox	Therapeutic vaccination	CEA, B7-1, ICAM-1, LFA-3	I	Stable disease in some patients	CEA-specific CTL	[75,76]
Vaccinia virus	Oncolytic virotherapy	GM-CSF	I	Not published	Not published	NCT01394939
Herpes simplex virus	Oncolytic virotherapy	None	I	Not published	Not published	NCT00149396
Adenovirus	Oncolytic virotherapy	None	I	Not published	Not published	NCT02028442

IL: interleukin; CEA: carcinoembryonic antigen; GUCY2C: guanylyl cylase C; Ep-CAM: epithelial cell adhesion molecule; ICAM-1: intercellular adhesion molecule; LFA: lymphocyte function-associated antigen; GM-CSF: granulocyte macrophage colony-stimulating factor.

**Table 2:** Oncolytic viroimmunotherapy for colon cancer in clinical trials.

Authors & Year	Vector	Phase	*N*	Delivery	Results	Adverse Effects	Immune Investigations
Kemeny & Fong 2006 [88] NCT00149396	NV1020 HSV+ GMCSF	I	12	3 × 10^6^ 3 × 10^7^ 1 × 10^8^	GGT rise, diarrhea, elev WBC		TNF-α, IL-2, IL-1, IFN-γ, CD4+/CD8+ ratio
Calvo 2014 [92] NCT02028442	Ad11/ad3 Enadenotucirev	I/II	161	1 × 10^10^–6 × 10^12^	No survival data reported yet	Flu-like sx, elevated GGT	Elevated TNF, IFN, IL-6, and IL-12 on Day 1 after higher doses
Park SH 2015 [89] NCT01380600	JX-594 tk attenuated Vaccinia	Ib	15	Up to 4 IV q14 days Dose 1 × 10^6^ pfu/kg, 1 × 10^7^, 3 × 10^7^	67% stable disease	Pox skin lesions Flu like symptoms	IL-2, IL-6, IL-8, IL-10, IL-18, MIP-1α, MCP-1, MIP-1β, and TNF-α
Balint 2015 [90] NCT02028442	A11/Ad3 group B adenovirus	I/II	32	1 × 10^9^ q3 weeks × 3 1 × 10^10^ q3 weeks × 3 1 × 10^11^ q3 weeks × 3 5 × 10^11^ q3 weeks × 3	No objective ant-tumor responses; Median survival 13mos in optimal tx grp	Injection site rxn Fever, flu-like symptoms	Cytolytic T cell responses IFN-γ TNF-α
NCT01274624	Reolysin + Folfiri + avastin in Folfiri naïve KRAS mutants	I	12	No data reported yet Due Fall 2017			
NCT02636036	Ad11/Ad3 Enadenotucirev + Anti-PD-1	I		Study completion June 2019			
